# Genetic Association Reveals Protection against Recurrence of *Clostridium difficile* Infection with Bezlotoxumab Treatment

**DOI:** 10.1128/mSphere.00232-20

**Published:** 2020-05-06

**Authors:** Judong Shen, Devan V. Mehrotra, Mary Beth Dorr, Zhen Zeng, Junhua Li, Xun Xu, David Nickle, Emily R. Holzinger, Aparna Chhibber, Mark H. Wilcox, Rebecca L. Blanchard, Peter M. Shaw

**Affiliations:** a Merck & Co., Inc., Kenilworth, New Jersey, USA; b BGI-Shenzhen, Shenzhen, China; c Shenzhen Key Laboratory of Unknown Pathogen Identification, Shenzhen, China; d School of Bioscience and Biotechnology, South China University of Technology, Guangzhou, China; e Leeds Teaching Hospital and University of Leeds, Leeds, United Kingdom; University of Michigan—Ann Arbor

**Keywords:** *Clostridium difficile*, antibacterials, bezlotoxumab, genomics

## Abstract

Bezlotoxumab is a human monoclonal antibody against Clostridium difficile toxin B, indicated to prevent recurrence of C. difficile infection (rCDI) in high-risk adults receiving antibacterial treatment for CDI. An exploratory genome-wide association study investigated whether human genetic variation influences bezlotoxumab response. DNA from 704 participants who achieved initial clinical cure in the phase 3 MODIFY I/II trials was genotyped. Single nucleotide polymorphisms (SNPs) and human leukocyte antigen (HLA) imputation were performed using IMPUTE2 and HIBAG, respectively. A joint test of genotype and genotype-by-treatment interaction in a logistic regression model was used to screen genetic variants associated with response to bezlotoxumab. The SNP *rs2516513* and the HLA alleles HLA-DRB1*07:01 and HLA-DQA1*02:01, located in the extended major histocompatibility complex on chromosome 6, were associated with the reduction of rCDI in bezlotoxumab-treated participants. Carriage of a minor allele (homozygous or heterozygous) at any of the identified loci was related to a larger difference in the proportion of participants experiencing rCDI versus placebo; the effect was most prominent in the subgroup at high baseline risk for rCDI. Genotypes associated with an improved bezlotoxumab response showed no association with rCDI in the placebo cohort. These data suggest that a host-driven, immunological mechanism may impact bezlotoxumab response. Trial registration numbers are as follows: NCT01241552 (MODIFY I) and NCT01513239 (MODIFY II).

**IMPORTANCE** Clostridium difficile infection is associated with significant clinical morbidity and mortality; antibacterial treatments are effective, but recurrence of C. difficile infection is common. In this genome-wide association study, we explored whether host genetic variability affected treatment responses to bezlotoxumab, a human monoclonal antibody that binds C. difficile toxin B and is indicated for the prevention of recurrent C. difficile infection. Using data from the MODIFY I/II phase 3 clinical trials, we identified three genetic variants associated with reduced rates of C. difficile infection recurrence in bezlotoxumab-treated participants. The effects were most pronounced in participants at high risk of C. difficile infection recurrence. All three variants are located in the extended major histocompatibility complex on chromosome 6, suggesting the involvement of a host-driven immunological mechanism in the prevention of C. difficile infection recurrence.

## Abstract (precis)

Clostridium difficile infection is associated with significant clinical morbidity and mortality; antibacterial treatments are effective, but recurrence of C. difficile infection is common. In this genome-wide association study, we explored whether host genetic variability affected treatment responses to bezlotoxumab, a human monoclonal antibody that binds C. difficile toxin B and is indicated for the prevention of recurrent C. difficile infection. Using data from the MODIFY I/II phase 3 clinical trials, we identified three genetic variants associated with reduced rates of C. difficile infection recurrence in bezlotoxumab-treated participants. The effects were most pronounced in participants at high risk of C. difficile infection recurrence. All three variants are located in the extended major histocompatibility complex on chromosome 6, suggesting the involvement of a host-driven immunological mechanism in the prevention of C. difficile infection recurrence.

## INTRODUCTION

Clostridium difficile is a Gram-positive, spore-forming, toxigenic bacterium that overgrows in the large intestine due to treatment with broad-spectrum antibiotics or due to disruptions in the normal gastrointestinal microbiome (1). C. difficile toxins (A and B) cause inflammation of the colon and can increase rates of morbidity and life-threatening conditions, including severe diarrhea and pseudomembranous colitis (1). In recent years, there has been a substantial increase in morbidity and mortality related to C. difficile infection (CDI), with the Centers for Disease Control and Prevention in the United States reporting a 400% increase in deaths between 2000 and 2007 (2), in part due to the emergence of a more virulent C. difficile strain type (3).

Current antibacterial treatment for primary CDI includes the use of vancomycin or fidaxomicin (4) and is often successful, with initial clinical cure rates commonly over 80% (5, 6). However, following antibacterial treatment, up to 25% of patients experience a first recurrent CDI (rCDI) (5–8). Of patients who experience rCDI, approximately 40% will have a second rCDI (7). This high rate of recurrence has been attributed to intestinal microbiome dysbiosis following antibiotic treatment for CDI and the persistence of C. difficile spores largely unaffected by antibacterials (9, 10). Patients at increased risk for rCDI include those with a prior episode of CDI, with severe infection, aged 65 years or more, immunocompromised, receiving concomitant antibiotics for non-CDI infection, and with hypervirulent C. difficile strains (e.g., ribotypes 027, 078, and 244) (7, 8, 11–16).

The efficacy and safety of bezlotoxumab were evaluated in the MODIFY I/II phase 3 trials in participants receiving antibacterial drug treatment for primary or rCDI (17). A single, 10-mg/kg (of body weight) intravenous dose of bezlotoxumab produced a consistent reduction in rCDI over 12 weeks compared with placebo infusion (10% and 37.5% absolute and relative reduction, respectively) (17).

While genetic variants among C. difficile strains are known to influence virulence and risks for mortality (18), little is known of the effects of host genetic variations on CDI and specifically CDI treatment outcomes. Using data collected from the MODIFY I/II trials, an exploratory genome-wide association study (GWAS) was conducted to investigate whether genetic variants were associated with response to bezlotoxumab with the specific aim of identifying genetic predictive biomarkers that could enable identification of patient subpopulations who may have preferential benefit with bezlotoxumab treatment. Identification of genetic markers that can predict treatment response may also provide putative mechanistic insight into new disease biology around CDI.

## RESULTS

### Participants.

In total, 2,655 participants were enrolled in the MODIFY I/II trials, of whom 2,559 were included in the modified intention-to-treat (mITT) population (17). One thousand one participants who consented to genetic sampling and passed GWAS quality controls (QCs) were included as the pharmacogenetic (PGx) population. Of these, 704 participants who were randomized to bezlotoxumab-containing or placebo arms and achieved initial clinical cure were included in the PGx GWAS analyses. The baseline characteristics and rCDI-related risk factors of the pharmacogenetic and mITT populations from MODIFY I/II are listed in Table 1. The pharmacogenetic and mITT populations were generally similar. The majority of participants (>70%) had at least one risk factor for rCDI, although the proportion was slightly smaller in the PGx population. CDI-related outcomes were similar in the mITT and PGx populations when combining all treatment arms (Table 1) and for each treatment arm (see Table S1 in the supplemental material).

**TABLE 1:** Participant characteristics and CDI-related outcomes in the overall and pharmacogenetic populations^*d*^

Characteristic or outcome	Value for population^*c*^:	
	mITT (*n* = 2,559)	PGx (*n* = 1,001)
Baseline characteristic		
Age (yr)		
Mean (SD)	63.3 (17.6)	61.9 (17.4)
Median	66	64
Range	18–100	18–99
Sex, female	1,444 (56.4)	598 (59.7)
≥65 yr of age	1,358 (53.1)	489 (48.9)
≥1 CDI episodes in past 6 months	704 (27.5)	286 (28.6)
≥2 previous CDI episodes ever	363 (14.2)	170 (17.0)
Severe CDI (Zar score ≥2)^*a*^	420 (16.4)	121 (12.1)
Immunocompromised	549 (21.5)	170 (17.0)
Charlson comorbidity index ≥3	1,054 (41.2)	360 (36.0)
Albumin ≤2.5 g/dl	332 (13.0)	104 (10.4)
Ribotype 027, 078, or 244	337 (21.1)	119 (18.9)
Antibiotic use during ADT	852 (33.3)	296 (29.6)
Antibiotic use after ADT	782 (30.6)	275 (27.5)
≥1 risk factor for rCDI^*b*^	1,941 (75.8)	706 (70.5)
Outcome		
Initial clinical cure	1,814 (70.9)	704 (70.3)
rCDI	454 (25.0)	191 (27.1)

a Zar score based on (i) age of >60 years (1 point), (ii) body temperature of >38.3°C (1 point), (iii) albumin level of <2.5 g/dl (1 point), (iv) peripheral white blood cell count of >15,000 cells/μl within 48 h (1 point), (v) endoscopic evidence of pseudomembranous colitis (2 points), and (vi) treatment in an intensive care unit (2 points).

b Prespecified risk factors for rCDI included age of ≥65 years; ≥1 CDI episodes in past 6 months; Zar score of ≥2; immunocompromised; ribotype 027, 078, or 244; and antibiotic use during/after ADT.

c Data are presented as *n* (%) unless otherwise indicated.

d Abbreviations: ADT, antibacterial drug treatment for CDI; CDI, Clostridium difficile infection; mITT, modified intent-to-treat; PGx, pharmacogenetic population; rCDI, recurrent Clostridium difficile infection.

10.1128/mSphere.00232-20.4 TABLE S1 Proportion of participants who experienced rCDI in the overall MODIFY I/II population and pharmacogenetic population. *^a^n* is the number of participants who had recurrence, and *N* is the number of the population. ACT, actoxumab; BEZ, bezlotoxumab; PGx, pharmacogenetic population; rCDI, recurrent Clostridium difficile infection. Download Table S1, DOCX file, 0.03 MB. Copyright © 2020 Shen et al. 2020 Shen et al. This content is distributed under the terms of the Creative Commons Attribution 4.0 International license.

### HLA association analysis.

Human leukocyte antigen (HLA) imputation identified two class II alleles, HLA-DRB1*07:01 and HLA-DQA1*02:01, in high linkage disequilibrium (LD, *r*^2^ = 0.98) that were also associated with bezlotoxumab treatment response to rCDI. HLA-DRB1*07:01 (minor allele frequency [MAF] = 0.10) was associated with rCDI (*P = *1.93 × 10^−5^) such that the per-allele odds ratio (OR) (95% confidence interval [CI]) was 0.19 (0.06 to 0.44). Similarly, HLA-DQA1*02:01 (MAF = 0.11) was associated with rCDI (*P = *5.18 × 10^−5^) with a per-allele OR (95% CI) of 0.21 (0.08 to 0.46) (Table 2).

**TABLE 2:** GWAS and HLA association results^*c*^

SNP/HLA allele	Chr	MAF	*n*	Overall *P* value^*a*^	BEZ and BEZ + ACT			Placebo		
					*P* value^*b*^	β (SE)	OR (95% CI)	*P* value^*b*^	β (SE)	OR (95% CI)
*rs2516513*	6	0.23	701	3.04 × 10^−08^	6.46 × 10^−08^	−1.19 (0.25)	0.31 (0.18–0.48)	6.97 × 10^−01^	−0.09 (0.23)	0.91 (0.57–1.44)
HLA-DRB1*07:01	6	0.10	689	1.93 × 10^−05^	1.65 × 10^−05^	−1.67 (0.48)	0.19 (0.06–0.44)	5.88 × 10^−01^	0.17 (0.32)	1.19 (0.63–2.21)
HLA-DQA1*02:01	6	0.11	699	5.18 × 10^−05^	1.80 × 10^−05^	−1.56 (0.44)	0.21 (0.08–0.46)	5.47 × 10^−01^	0.19 (0.31)	1.21 (0.65–2.21)

a Overall *P* value (*P* value from joint test of genotype and genotype-by-treatment interaction).

b *P* value from 1-df test of SNP.

c Abbreviations: ACT, actoxumab; BEZ, bezlotoxumab; Chr, chromosome; CI, confidence interval; df, degree of freedom; GWAS, genome-wide association study; HLA, human leukocyte antigen; MAF, minor allele frequency; OR, odds ratio; SNP, single nucleotide polymorphism.

### Genome-wide association analysis.

**FIG 1:**
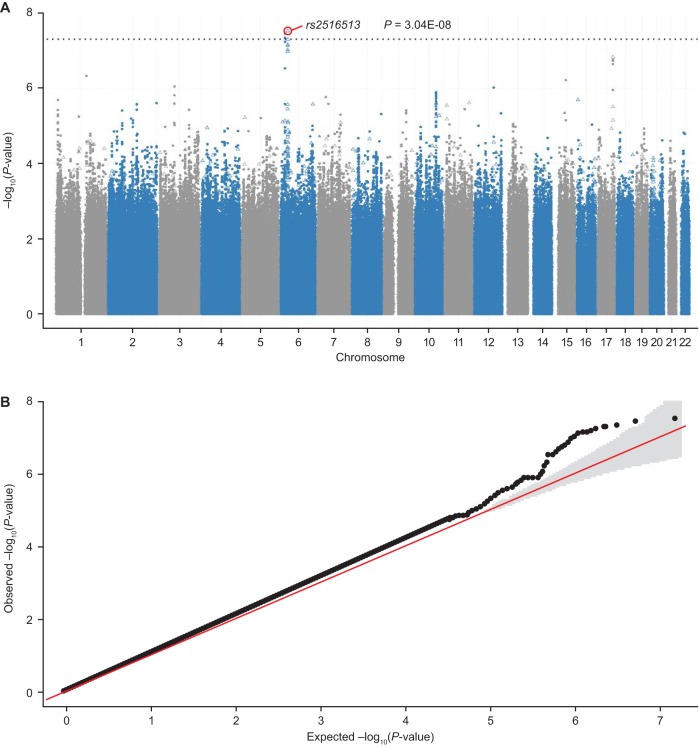
Manhattan plot (A) and QQ plot (B) showing the significance of SNP *rs2516513* associated with drug-induced reduction on rCDI in the GWAS analysis (placebo arm versus bezlotoxumab and bezlotoxumab + actoxumab arms). λ_GC_ is 1.06 in the QQ plot. Open triangles represent the assayed SNPs; solid symbols represent the imputed SNPs. The dotted line is the genome-wide significance *P* value threshold of 5 × 10^−08^. GWAS, genome-wide association study; rCDI, recurrent Clostridium difficile infection; SNP, single nucleotide polymorphism.

**FIG 2:**
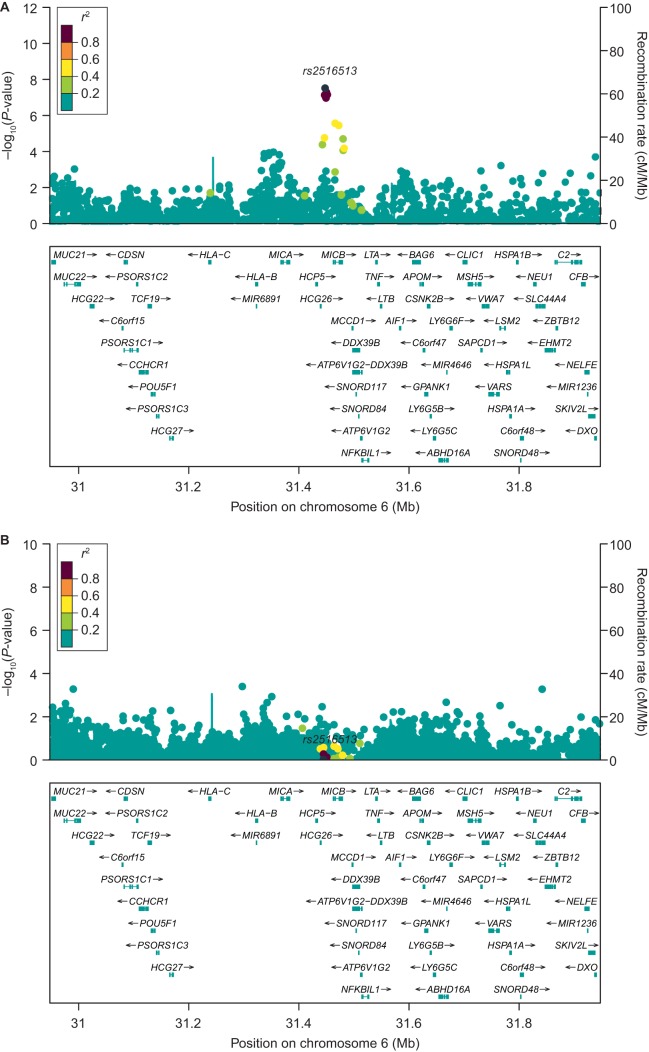
Regional association plot of 500 kb on each side of the *rs2516513* SNP before (A) and after (B) conditioning. SNP, single nucleotide polymorphism.

After single nucleotide polymorphism (SNP) imputation and QC, there were 7,570,264 variants available for GWAS analysis. The common intergenic SNP *rs2516513* (6:31447588, MAF = 0.23) was associated with a reduction in rCDI in bezlotoxumab-treated participants (*P = *6.46 × 10***^−^***^8^; per-allele OR, 0.31; 95% CI, 0.18 to 0.48) (Table 2) but not in placebo-treated participants (per-allele OR, 0.91; 95% CI, 0.57 to 1.44). The SNP *rs2516513* joint test of genotype and genotype-by-treatment interaction *P* value was 3.04 × 10***^−^***^8^, and the genotype-by-treatment interaction *P* value was 4.44 × 10***^−^***^5^. Manhattan and QQ plots from the GWAS analysis are shown in Fig. 1. SNP *rs2516513* is located between the HCP5 and MICB genes in the extended major histocompatibility complex (xMHC) on chromosome 6, as shown in Fig. 2A. The *rs2516513* T allele was carried by 41% of participants in the clinical trial population, consistent with the allele frequency of this SNP in individuals of European descent in the 1000 Genomes database (19). In addition to *rs2516513*, two other SNPs in high LD (*r*^2^ = 0.99) were also associated with a reduction in rCDI rate in bezlotoxumab-treated participants: *rs113379306* (6:17333351, MAF = 0.04; *P = *3.54 × 10***^−^***^8^) and *rs76166871* (6:17329940, MAF = 0.04; *P = *4.64 × 10***^−^***^8^). However, after conditioning on *rs2516513*, these SNPs were not associated with rCDI risk in bezlotoxumab-treated participants (Fig. 2B), indicating that SNP *rs2516513* was the primary signal in this region. There was weak LD (*r*^2^ = 0.14) between *rs2516513* and HLA-DRB1*07:01.

### Assessment of evidence for causal variant and gene.

Fine-mapping of the GWAS summary statistics at the *rs2516513* locus identified 26 variants with posterior probability of causality of >1%, including the lead variant *rs2516513.* These variants are all noncoding; therefore, we examined published gene expression, protein expression, and methylation data to identify a potential causal gene(s) for the GWAS signal. To do this, we assessed whether the lead GWAS SNP or any SNP in high LD (*r*^2^ > 0.8) was a significant expression quantitative trait locus (eQTL), protein quantitative trait locus (pQTL), or methylation quantitative trait locus (meth-QTL). These variants were linked to 26 genes by at least one of the data sources used (Table S2). Three genes were linked to the locus by at least two of the data sources used: *MICB*, *HCG27*, and *C4B.* Further details regarding the cell or tissue types and directionality of association for these three genes are included in Table S3.

10.1128/mSphere.00232-20.5 TABLE S2 Putative causal gene analysis at *rs2516513* locus. Analysis evaluated whether *rs2516513* or variants in high LD (*r*^2^ > 0.8) are significant eQTLs, pQTLs, or meth-QTLs. Shaded rows indicate genes that were linked to the lead SNP (or a high LD proxy) in two or more sources. Download Table S2, DOCX file, 0.04 MB. Copyright © 2020 Shen et al. 2020 Shen et al. This content is distributed under the terms of the Creative Commons Attribution 4.0 International license.

10.1128/mSphere.00232-20.6 TABLE S3 Functional annotations for *rs2516513*. Associations in reference to *rs2516513*-T allele. Arrow indicates direction of effect (i.e., increased or decreased expression/methylation associated with T allele). Download Table S3, DOCX file, 0.04 MB. Copyright © 2020 Shen et al. 2020 Shen et al. This content is distributed under the terms of the Creative Commons Attribution 4.0 International license.

### Conditional association analysis.

Conditional regression analyses further showed that the signals from HLA-DRB1*07:01 and HLA-DQA1*02:01 were driven in large part by signals from *rs2516513*. After conditioning on HLA-DRB1*07:01 and HLA-DQA1*02:01, pairwise association *P* values for *rs2516513* were 5.70 × 10^−5^ and 1.42 × 10^−5^, respectively. The conditional association results of HLA-DRB1*07:01 and HLA-DQA1*02:01 were similar, with *P* values of 0.014 and 0.037 (both <0.05), respectively, while conditioning on *rs2516513*, since they are in high LD with each other.

### rCDI summarized by genotype and risk category.

**FIG 3:**
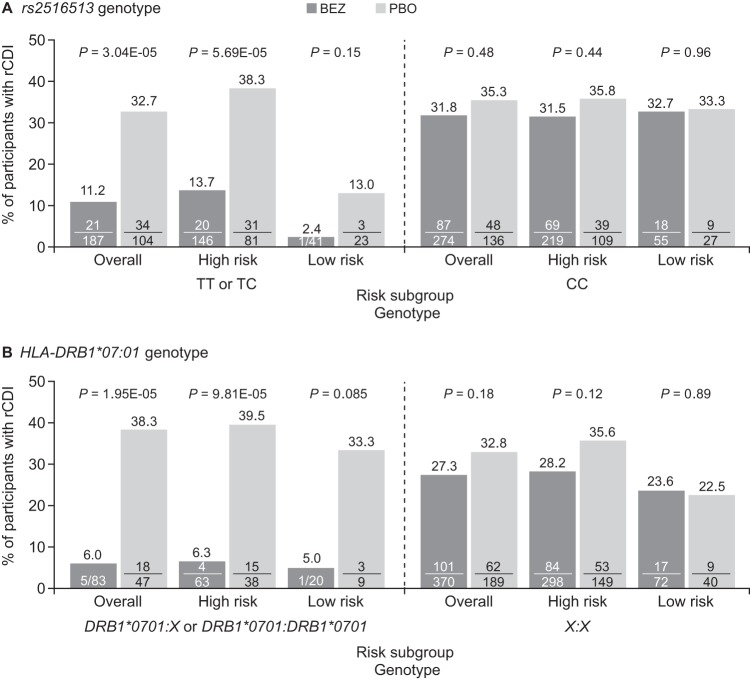
Proportion of participants with rCDI stratified by genotype and risk category. (A) *rs2516513* genotype. (B) HLA-DRB1*07:01 genotype. The high-risk subgroup included participants with one or more of the following factors: prior episode of CDI in the past 6 months, severe CDI at baseline (per Zar score [37]), age of ≥65 years, CDI due to a hypervirulent strain (027, 078, or 244 ribotypes), immunocompromised, or receiving concomitant systemic antibiotics. Participants at low risk of rCDI were those with none of the above risk factors. *P* values were calculated from two-sided Fisher’s exact tests. BEZ, bezlotoxumab; CDI, Clostridium difficile infection; HLA, human leukocyte antigen; PBO, placebo; rCDI, recurrent Clostridium difficile infection.

Demographic and clinical rCDI risk factors were similar between participants with TT or TC genotypes (SNP^+^) and those with CC genotypes (SNP^−^) and between participants with HLA-DRB1*07:01:HLA-DRB1*07:01 or HLA-DRB1*07:01:X (HLA^+^) and *X:X* (HLA^−^) genotypes (Table S4). In participants treated with bezlotoxumab who carried the T allele of SNP *rs2516513* (i.e., TC or TT genotype), the rate of rCDI was reduced compared with participants in the placebo group (−21.5% absolute difference; two-sided Fisher’s exact test, *P = *3.04 × 10***^−^***^5^) (Fig. 3A and Table S5). The effect of the T allele was most prominent in the subgroup of bezlotoxumab-treated participants at high risk of rCDI (−24.6% absolute difference versus placebo; two-sided Fisher’s exact test, *P = *5.69 × 10***^−^***^5^). This trend was less pronounced in the low-risk subgroup (−10.6% absolute difference versus placebo, two-sided Fisher’s exact test, *P = *0.15), which may be due to the low number (64) of participants and the low rate (6.25%) of rCDI in this subgroup. In CC homozygous participants, rCDI rates exceeded 30% in both treatment groups and in participants at high and low risk of rCDI (Fig. 3A).

10.1128/mSphere.00232-20.7 TABLE S4 Participant characteristics of *rs2516513* SNP^+^ (TC or TT genotypes) and SNP^−^ (CC genotypes) subgroups and HLA-DRB1*07:01 HLA^+^ (DRB1*07:01:DRB1*07:01 or DRB1*07:01:X) and HLA^−^ (*X:X*) subgroups. Data are presented as *n* (%) unless otherwise indicated. *^a^*Zar score based on (i) age of >60 years (1 point), (ii) body temperature of >38.3°C (1 point), (iii) albumin level of <2.5 g/dl (1 point), (iv) peripheral white blood cell count of >15,000 cells/μl within 48 h (1 point), (v) endoscopic evidence of pseudomembranous colitis (2 points), and (vi) treatment in an intensive care unit (2 points). *^b^*Prespecified risk factors for rCDI included age of ≥65 years; ≥1 CDI episodes in past 6 months; Zar score of ≥2; immunocompromised; 027, 078, or 244 strain; antibiotic use during ADT; and antibiotic use after ADT. Abbreviations: ADT, antibacterial drug treatment for CDI; CDI, Clostridium difficile infection; HLA, human leukocyte antigen; rCDI, recurrent Clostridium difficile infection; SNP, single nucleotide polymorphism. Download Table S4, DOCX file, 0.03 MB. Copyright © 2020 Shen et al. 2020 Shen et al. This content is distributed under the terms of the Creative Commons Attribution 4.0 International license.

10.1128/mSphere.00232-20.8 TABLE S5 Proportion of participants with rCDI stratified by the *rs2516513* genotype subgroups in the total and genetically defined Caucasian populations. Risk difference = (BEZ and BEZ+ACT) − PBO. Relative risk = (BEZ and BEZ+ACT)/PBO. Note: 59% CC carriers and 41% TC and TT carriers (57% CC and 43% TC or TT in genetically defined Caucasian population) in phase 3 studies (MODIFY I + MODIFY II). Download Table S5, DOCX file, 0.03 MB. Copyright © 2020 Shen et al. 2020 Shen et al. This content is distributed under the terms of the Creative Commons Attribution 4.0 International license.

Bezlotoxumab-treated participants who carried at least one HLA-DRB1*07:01 allele also had a reduced rate of rCDI compared with placebo treatment (−32.3% absolute difference; two-sided Fisher’s exact test, *P = *1.95 × 10^−5^). This effect was also observed in the high-risk subgroup (−33.2% absolute difference versus placebo; two-sided Fisher’s exact test, *P = *9.81 × 10^−5^) but not in the low-risk subgroup (−28.3% absolute difference versus placebo; two-sided Fisher’s exact test, *P = *0.085). Among noncarriers of the HLA-DRB1*07:01 allele, no treatment differences in rCDI were observed in the overall group or in high- or low-risk subgroups (Fig. 3B and Table S6).

10.1128/mSphere.00232-20.9 TABLE S6 Proportion of participants with rCDI stratified by the HLA-DRB1*07:01 genotype subgroups in the total and genetically defined Caucasian populations. Risk difference = (BEZ and BEZ+ACT) − PBO. Relative risk = (BEZ and BEZ+ACT)/PBO. Note: 81% X:X carriers and 19% HLA-DRB1*07:01 allele carriers (HLA-DRB1*07:01:X or HLA-DRB1*07:01:HLA-DRB1*07:01) in phase 3 studies (MODIFY I + MODIFY II). In generically defined Caucasian population 79% XX and 21% HLA-DRB1*07:01:X or HLA-DRB1*07:01:HLA-DRB1*07:01 in phase 3 studies (MODIFY I + MODIFY II). Download Table S6, DOCX file, 0.03 MB. Copyright © 2020 Shen et al. 2020 Shen et al. This content is distributed under the terms of the Creative Commons Attribution 4.0 International license.

**FIG 4:**
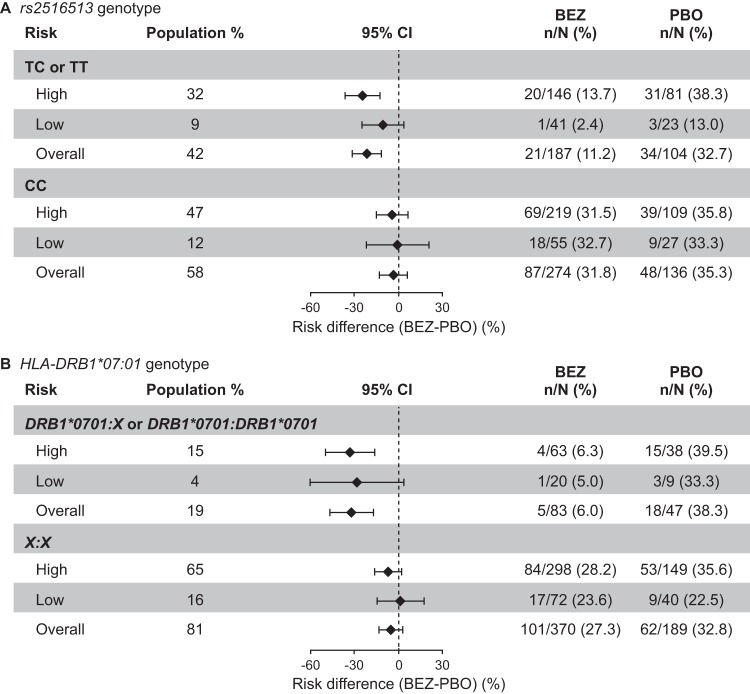
CDI recurrence stratified by genotypes and rCDI risk categories: *rs2516513* genotype (A) and HLA-DRB1*07:01 genotype (B). The high-risk subgroup included participants with one or more of the following factors: prior episode of CDI in the past 6 months, severe CDI at baseline (per Zar score [37]), age of ≥65 years, CDI due to a hypervirulent strain (027, 078, or 244 ribotypes), immunocompromised, or receiving concomitant systemic antibiotics. Participants at low risk of rCDI were those with none of the above risk factors. BEZ, bezlotoxumab; CDI, Clostridium difficile infection; CI, confidence interval; PBO, placebo; rCDI, recurrent Clostridium difficile infection.

As shown in Fig. 4, the reduction in risk of rCDI following bezlotoxumab treatment versus placebo varied depending on the baseline risk category. Participants carrying the *rs2516513* T allele or the HLA-DRB1*07:01 allele exhibited a strong trend for benefit of bezlotoxumab treatment in the high-risk rCDI category. In contrast, noncarriers of the *rs2516513* T allele or the HLA-DRB1*07:01 allele showed limited benefit from bezlotoxumab treatment. Because only a small percentage of these participants were in the low-risk subgroups (*rs2516513* T allele, 64/701 [9%]; HLA-DRB1*07:01 allele, 29/689 [4%]), the CIs are wide and cross zero.

## DISCUSSION

These findings highlight the potential value of conducting GWAS analyses in phase 3 studies. Identifying a genetic signal during clinical development provides an early indication of a potential treatment-predictive biomarker; furthermore, such a finding allows for consideration of possible routes for validation and for recognition of potential new mechanisms that might enable further improvements in therapeutic intervention.

This exploratory GWAS identified the T allele carriers of SNP variant *rs2516513*, and HLA carriers of the alleles HLA-DRB1*07:01 and HLA-DQA1*02:01, as being associated with a positive treatment response to bezlotoxumab relative to placebo (reduced rCDI rate among allele carriers). The effects were most pronounced in participants determined to be at high risk for rCDI based on clinical factors used to determine likelihood for rCDI. A smaller effect was seen in bezlotoxumab-treated participants at low risk for rCDI with the same trend; however, statistical significance was not reached, possibly due to the low numbers of participants in the low-risk category. In addition, bezlotoxumab treatment responses in participants carrying both the SNP *rs2516513* T allele and the HLA-DRB1*07:01 allele were similar to those from carriers of each individual risk allele (see Table S7 in the supplemental material). Furthermore, results from GWAS sensitivity analysis using Caucasian-only participants were very similar to those from all participants (Tables S5 and S6).

10.1128/mSphere.00232-20.10 TABLE S7 Proportion of participants with rCDI stratified by the *rs2516513 *+ HLA-DRB1*07:01 composite genotype subgroups. Risk difference = (BEZ and BEZ+ACT) − PBO. Relative risk = (BEZ and BEZ+ACT)/PBO. Note: 55% CC or X:X and 45% TC or TT or HLA-DRB1*07:01:X or HLA-DRB1*07:01:HLA-DRB1*07:01 in phase 3 studies (MODIFY I + MODIFY II). Download Table S7, DOCX file, 0.03 MB. Copyright © 2020 Shen et al. 2020 Shen et al. This content is distributed under the terms of the Creative Commons Attribution 4.0 International license.

There was no correlation between clinical risk factors and genetic risk factors, as easily ascertained risk factors for rCDI that were collected during the clinical trial were similar in the SNP^+^ (*rs2516513* TC or TT genotypes) and SNP^−^ (*rs2516513*, CC genotypes) subgroups and the HLA^+^ (HLA-DRB1*07:*01*:HLA-DRB1*07:01 or HLA-DRB1*07:01:X) and HLA^−^ (*X:X*) subgroups (Table S4). This indicates that currently known clinical and demographic risk factors for rCDI are not likely to be useful as a surrogate to easily define the genotype subgroups (for example, SNP^+^ or HLA^+^ genotype subgroups) who would benefit from bezlotoxumab treatment.

While different baseline patient characteristics and variation in C. difficile strains are known to influence the incidence of rCDI (7, 8, 11, 14, 18), there have been few studies on the effects of host genetic polymorphisms on rCDI. One recent study of data collected during a C. difficile outbreak at a tertiary care center found that host factors were more important predictors for rCDI than strain type or use of antibiotics (20). Moreover, an earlier prospective cohort study found that a common polymorphism in the interleukin-8 promoter region was associated with an increased risk for rCDI, with participants carrying the AA allele having an approximately 2-fold-greater risk of rCDI than participants with AT or TT genotypes (21). However, we and others were unable to replicate the interleukin-8 promoter finding in our study (see the supplemental material) (22). Another study investigating primary toxigenic C. difficile colonization found that a polymorphism in the Toll-like receptor 4, *rs1927914*, was independently associated with colonization (23). Taken together, the previous studies suggest that use of host genetic profiling may identify participants at high risk for rCDI. However, in this study our findings suggest that we have identified genetic factors specifically associated with bezlotoxumab treatment response (i.e., with a strong predictive treatment effect), which are not associated with rCDI (i.e., with prognostic effects related to CDI recurrence).

The identification of bezlotoxumab treatment-associated genetic loci on chromosome 6 within the xMHC suggests the potential involvement of a host-driven, immunological mechanism in response to bezlotoxumab treatment of rCDI. An intergenic variant in the xMHC, *rs2516513*, was associated with treatment benefit. In other studies, the SNP *rs2516513* has been shown to associate with HIV-1 controllers and progressors (24), suggesting that it may contribute to immune control of HIV. *rs2516513* or variants in high LD have been linked to mRNA or protein expression or methylation level of a number of genes. For example, *rs2516513* has been linked to increase in protein expression of *MICB* in human plasma and mRNA expression in CD14^+^ monocytes, as well as decreased expression in naive CD4^+^ T cells and adipose, thyroid, and skin tissues (see Table S3 in the supplemental material). *MICB* encodes MHC class I polypeptide-related sequence B (MICB), a cell surface protein expressed in response to stress that is recognized by certain types of T cells and natural killer cells (25). While the function of MICB is not known, there is evidence that it plays a role in immune response to pathogens (26). *rs2516513* is also associated with a decrease in mRNA expression of *C4B*, encoding complement component 4B, in naive CD4 T cells and an increase in plasma protein levels (Table S3); the complement system plays a key role in innate immune response. While the most likely causal genes at the locus are linked to immune response and no association was observed in the placebo arm, further investigation is required to determine the mechanism linking change in function of the causal gene specifically to treatment response. More broadly, the *rs2516513* locus can be linked to a number of other genes, and further studies would be required to determine conclusively the causal gene driving the association between this locus and response to bezlotoxumab.

Two HLA alleles, HLA-DRB1*07:01 and HLA-DQA1*02:01, were also associated with treatment benefit. HLA molecules play a pivotal role in the adaptive immune response, binding peptide fragments from pathogens and displaying them on the cell surface for recognition by T cells (27). They are also known for having a high level of genetic polymorphisms, which likely enable the host to respond to a range of different and rapidly evolving pathogens (27). Previous studies have indicated a link between adaptive immune response to infection and protection from rCDI, with a serum antibody response to C. difficile toxins being associated with subsequent protection from recurrence (28). Furthermore, a study in MHC class II knockout mice and CD4^+^ T-cell knockout mice found that protection from rCDI is dependent on antitoxin antibody formation and requires MHC cass II genes (29). Bezlotoxumab has low immunogenicity potential (30), suggesting that the association between specific HLA alleles and response to treatment is not related to an immunogenic response against bezlotoxumab itself, but rather a host response against infection that becomes important specifically in subjects administered treatment. However, conditional analysis indicates that the association with HLA alleles may be largely driven by the *rs2516513* locus, rather than by an independent association driven by a particular HLA allele.

As the results of this study are purely exploratory, the findings cannot be considered conclusive and require confirmation in an independent validation study. If these loci are confirmed as predictive for response to bezlotoxumab, future research should also focus on the mechanism underlying this effect.

In conclusion, this exploratory GWAS identified an SNP (*rs2516513*) and two HLA alleles (HLA-DRB1*07:01 and HLA-DQA1*02:01) in the xMHC region on chromosome 6 associated with an approximately 2-fold- and 3-fold-decreased risk for rCDI in bezlotoxumab-treated participants, respectively. These effects were most prominent in participants at high risk for rCDI carrying either the *rs2516513* T allele or the HLA-DRB1*07:01 allele. A smaller (non-statistically significant) effect was seen in participants at low risk for rCDI with the same trend. Further confirmation of these loci as predictors for rCDI prevention with bezlotoxumab treatment needs to be performed.

## MATERIALS AND METHODS

### Study design.

MODIFY I (trial registration no. NCT01241552) and MODIFY II (trial registration no. NCT01513239) were randomized, double-blind, placebo-controlled, multicenter, phase 3 trials that were conducted from 1 November 2011 to 22 May 2015 at 322 sites in 30 countries (17). Full details of the studies have previously been published (17). Briefly, participants receiving oral metronidazole, vancomycin, or fidaxomicin for 10 to 14 days for the treatment of primary CDI or rCDI were randomized to receive an infusion of bezlotoxumab (10 mg/kg), actoxumab (10 mg/kg) (MODIFY I only), bezlotoxumab plus actoxumab (10 mg/kg each), or placebo (0.9% saline). CDI was defined as ≥3 unformed bowel movements (types 5 to 7 on the Bristol stool scale [31] in 24 h) with a stool test positive for toxigenic C. difficile. Occurrence of rCDI within 12 weeks of follow-up was assessed in participants who achieved an initial clinical cure, which was defined as no diarrhea during the two consecutive days following completion of ≤16 calendar days of antibacterial drug treatment for CDI. rCDI was defined as a new episode of CDI after initial clinical cure of the baseline episode.

MODIFY I and II were conducted in accordance with Good Clinical Practice guidelines and the provisions of the Declaration of Helsinki. The protocols and amendments were approved by the institutional review board or independent ethics committee at each study site. Written informed consent was provided by all participants before the trial began.

### Genotyping, QC, and imputation.

DNA was extracted from peripheral blood samples collected from participants in MODIFY I/II who consented to PGx analyses. Genotyping was performed using an Axiom array platform (Affymetrix Axiom array); genotype imputation was performed using the 1000 Genomes phase 1 reference data and IMPUTE2 software (32), after standard GWAS QC but prior to the genetic analysis. The details of the GWAS QC and SNP imputation are described in Text S1 and Fig. S1 in the supplemental material. The HLA alleles in three class I loci (*HLA-A*, *HLA-B*, and *HLA*-*C*) and four class II loci (*HLA-DRB1*, *HLA-DQA1*, *HLA-DQB1*, and *HLA-DPB1*) were prespecified for the association analysis and were imputed using HLA Genotype Imputation with Attribute Bagging (HIBAG) (33). The best-guess imputed HLA types were used by setting the call rate threshold to 0.5, meaning that the imputed genotypes were set as missing if their imputation posterior probability was less than 0.5. The multiallelic HLA types were converted to the biallelic HLA alleles for each unique HLA allele. The biallelic HLA alleles were then recoded as 0, 1, and 2 to reflect the number of minor alleles carried by participants. For example, for the allele HLA-A*11:01, genotypes *X/X*, *HLA*-A*11:01/X, and HLA-A*11:01/HLA-A*11:01 would be assigned as 0, 1, and 2, respectively. In total, 219 HLA alleles from three class I and four class II HLA genes were imputed via HIBAG.

10.1128/mSphere.00232-20.1 TEXT S1 Supplemental methods: GWAS quality control, SNP/HLA imputation, and PC analysis. Download Text S1, DOCX file, 0.04 MB. Copyright © 2020 Shen et al. 2020 Shen et al. This content is distributed under the terms of the Creative Commons Attribution 4.0 International license.

10.1128/mSphere.00232-20.2 FIG S1 C. difficile GWAS variant-level (a) and subject-level (b) QC workflow. Download FIG S1, DOCX file, 0.1 MB. Copyright © 2020 Shen et al. 2020 Shen et al. This content is distributed under the terms of the Creative Commons Attribution 4.0 International license.

### Statistical analysis.

In PGx studies, a patient’s clinical outcomes are influenced by both prognostic and predictive factors. A prognostic biomarker discovered by testing the genotype main effect affects the likelihood of the clinical phenotype regardless of the type of treatment, which is useful in classifying patients into different risk categories indicating the condition of the disease. In contrast, a predictive biomarker discovered by testing the genotype-by-treatment interaction affects the likelihood of the clinical event for a treatment, which is useful in segmenting patients into treatment response and nonresponse groups. The joint test of the main genetic effect and the genotype-by-treatment interaction effect usually increases power for detecting signals in PGx studies compared with only testing the interaction effect or the main genotype effect separately (45). Thus, we used the 2-degree-of-freedom (2-df) likelihood test (joint test of genotype and genotype-by-treatment interaction) *P* value as the screen step to assess the combined prognostic and (treatment-related) predictive association of each genetic variant to drug response (rCDI) and declare statistical significance in this randomized clinical trial GWAS analysis with small sample size (*n* = 704). In addition, we also generated the 1-df test of the main genotype *P* value and the 1-df test of the main genotype-by-treatment interaction *P* value, the 1-df test *P* value in the treatment (bezlotoxumab [BEZ] and BEZ + actoxumab [ACT]) arm, and the 1-df test *P* value in the placebo arm to help interpret the results.

To provide increased statistical power for the exploratory GWAS, data from the bezlotoxumab-containing arms and placebo arms, respectively, were pooled across both MODIFY I/II trials. The actoxumab-alone arm was not used in this analysis. Genetic principal components (PCs) were calculated using EIGENSOFT (34); the first five PCs were used as covariates in the statistical models to control for confounding due to population stratification in the samples, which included participants from multiple race groups (88.5% were Caucasian). The five top PCs were determined from the scree plot since they explain most of the variance (Fig. S2). The PC analysis steps are summarized in Text S1. Other covariates such as hospitalization flag (inpatient or outpatient at the time of randomization into the trial [HOSPSTR]) and antibacterial drug treatment for CDI (ADT) flag (fidaxomicin, metronidazole, or vancomycin [ADTSTR]) were included in the models, and the genotypes were coded to detect additive genetic effects. Within each genetic variant, genotype was numerically coded for an individual participant as 0, 1, or 2 to reflect the number of copies of the minor allele. Treatment was numerically recoded as 0, 1, and 2 depending on whether, in addition to ADT, a participant received placebo, monotherapy (bezlotoxumab alone), or combination therapy (actoxumab plus bezlotoxumab), respectively, since the clinical efficacy results show a reduction of rCDI rates versus placebo while monotherapy and combination therapy have an increasing or additive trend (17).

10.1128/mSphere.00232-20.3 FIG S2 C. difficile GWAS PC analysis plots. Download FIG S2, DOCX file, 0.1 MB. Copyright © 2020 Shen et al. 2020 Shen et al. This content is distributed under the terms of the Creative Commons Attribution 4.0 International license.

The full statistical model was logit(*p_i_*) = β_0_ + β_cov_*X_i_* + β_1_ × trt*_i_* + β_2_ × *g_i_* + β_3_ × trt*_i_* × *g_i_*, where *p_i_* is the CDI recurrence rate for genotype or subject *i* and the *X_i_* are subject *i*’s covariates (multiple covariates) including HOSPSTR, ADTSTR, and PC1 to PC5. The comparative statistical model was logit(*p_i_*) = β_0_ + β_cov_*X_i_* + β_1_ × trt*_i_*.

A standard 2-df likelihood ratio-based test in a logistic regression model was used to test the joint null hypothesis of no genotype main effect and no genotype-by-treatment interaction. Due to the limited power of SNPs with low MAF, 7,570,264 SNPs with MAF of ≥1% were tested in this analysis. A standard Bonferroni correction assuming 1 million independent SNPs was used for multiplicity adjustment, so that SNPs with *P < *5 × 10^−8^ were considered to be genome-wide statistically significant in the context of a maximum familywise type I error rate of 5%. In addition to the 2-df test, we also conducted the 1-df tests in the treatment (BEZ and BEZ + ACT) arms and placebo arm separately to help interpret the overall association results. The full statistical model and the comparative statistical model are the same as described above except that the treatment term and the genotype-by-treatment interaction term should be dropped in the full statistical model and the treatment term should be dropped in the comparative statistical model.

Prior studies have indicated a link between immune response and protection from rCDI, with a serum antibody response to C. difficile toxins being associated with subsequent protection from recurrence (28). Furthermore, a study in MHC class II knockout mice and CD4^+^ T-cell knockout mice found that protection from rCDI requires MHC class II genes and is dependent on T-cell help (29). Because of the prior evidence linking immune response with rCDI, we conducted HLA association analyses in the xMHC as a separate candidate gene study including the analysis of the three class I genes (*HLA-A*, *HLA-B*, and *HLA-C*) and four class II genes (*HLA-DRB1*, *HLA-DQA1*, *HLA-DQB1*, and *HLA-DPB1*). The statistical association analysis method for HLA alleles was the same as that described above for GWAS SNPs. A total of 112 out of 219 imputed HLA alleles with MAF of ≥1% were tested in this analysis. Bonferroni correction was used for multiplicity adjustment, and the *P* value threshold for statistical significance was set at 4.46 × 10^−4^ (0.05/112) for HLA association analysis.

For SNPs or HLA alleles declared statistically significant with the 2-df joint test, a logistic regression-based likelihood ratio test with 1 df was conducted separately in the bezlotoxumab-containing and placebo arms to assess the association between rCDI and genotype. Effect sizes and ORs were further reported. All statistical analyses were performed using PLINK (35) and R (36).

### Subgroup analysis.

Because bezlotoxumab is indicated for patients at high risk for rCDI, the proportion of participants with rCDI was estimated for each treatment cohort by genotype and rCDI risk category. Participants considered at high risk of rCDI were defined as those having one or more of the following factors: prior episode of CDI in the past 6 months, severe CDI at baseline (per Zar score [37]), age of ≥65 years, CDI due to a hypervirulent strain (027, 078, or 244 ribotypes), immunocompromised, or receiving concomitant systemic antibiotics. Meanwhile, participants at low risk of rCDI were those with none of the above risk factors. The risk difference and its 95% CI were calculated in the subgroups stratified by minor allele/homozygote major allele carriers and high-/low-risk group of rCDI for comparison.

### Fine-mapping.

To determine the probability of each SNP in LD with lead SNP *rs2516513* being the causal variant at the locus, we ran a simple fine-mapping script in R using GWAS summary statistics. This method calculates approximate Bayes factors from effect sizes (betas) and standard errors (38). This method also assumes that there is one causal variant and places equal priors on all variants (39). Because the summary statistics were from a likelihood ratio test (LRT) with 2-df, we first converted the LRT *P* values to betas and standard errors using the method from the R package ‘coloc’ (40). The 95% credible set was very large (4,082 variants), likely due to the high number of variants in tight LD in this region. There were 26 variants with posterior probability of >1%, including the lead variant *rs2516513*.

### Causal gene analysis.

To determine the possible causal gene driving the association at the *rs2516513* locus, we assessed whether the lead GWAS SNP or any SNP in high LD (*r*^2^ > 0.8) was a significant eQTL, pQTL, or meth-QTL. Significance level was determined by each study, with the most commonly used threshold being false discovery rate (FDR) of <0.05. Details of significance level calculation and other analytical methods are provided in the indicated publications: eQTL data across 48 different tissues (41); eQTL data from CD14^+^ monocytes, CD16^+^ neutrophils, and naive CD4^+^ T cells (42); meth-QTLs for CD14^+^ monocytes, CD16^+^ neutrophils, and naive CD4^+^ T cells (genes identified that overlap methylated regions) (42); eQTL data from gamma interferon-stimulated primary monocytes (43); pQTL data from human plasma proteome (44).

### Data availability.

The data sharing policy of Merck Sharp & Dohme, including restrictions, is available at http://engagezone.msd.com/ds_documentation.php. Requests for access to the GWAS summary statistics results from this clinical study can be submitted through the EngageZone site or via email to dataaccess@merck.com.
