# Supplementary material for: Genetic Association Reveals Protection against Recurrence of Clostridium difficile Infection with Bezlotoxumab Treatment
Source: mSphere. 2020 May 6;5(3):e00232-20. doi: 10.1128/mSphere.00232-20 (PMC7203456; doi:10.1128/mSphere.00232-20)
Supplement: TABLE S1 [file mSphere.00232-20-st001.docx]

| Treatment | MODIFY I/II  n/N^a^ (%) | PGx  n/N^a^ (%) |
| --- | --- | --- |
| BEZ + ACT | 119/568 (21.0) | 50/223 (22.4) |
| BEZ | 129/625 (20.6) | 59/241 (24.5) |
| Placebo | 206/621 (33.2) | 82/240 (34.2) |
| Total | 454/1,814 (25.0) | 191/704 (27.1) |
