# Supplementary material for: Genetic Association Reveals Protection against Recurrence of Clostridium difficile Infection with Bezlotoxumab Treatment
Source: mSphere. 2020 May 6;5(3):e00232-20. doi: 10.1128/mSphere.00232-20 (PMC7203456; doi:10.1128/mSphere.00232-20)
Supplement: TABLE S2 [file mSphere.00232-20-st002.docx]

| Gene | eQTL  (GTEx V7) (3) | eQTL (Chen 2016) (4) | eQTL (FairFax 2014) (5) | meth-eQTL  (Chen 2016) (4) | pQTL  (Sun 2018) (6) | Sum |
| --- | --- | --- | --- | --- | --- | --- |
| MICB | X | X |  | X | X | 4 |
| HCG27 | X | X |  |  |  | 2 |
| C4B |  | X |  |  | X | 2 |
| HLA-C | X |  |  |  |  | 1 |
| PSORS1C1 | X |  |  |  |  | 1 |
| XXbac-BPG181B23.7 | X |  |  |  |  | 1 |
| HLA-S | X |  |  |  |  | 1 |
| MICA | X |  |  |  |  | 1 |
| MDC1 | X |  |  |  |  | 1 |
| ZBTB12 | X |  |  |  |  | 1 |
| XXbac-BPG299F13.17 | X |  |  |  |  | 1 |
| ATP6V1G2 | X |  |  |  |  | 1 |
| XXbac-BPG248L24.12 | X |  |  |  |  | 1 |
| HLA-B | X |  |  |  |  | 1 |
| PSORS1C2 | X |  |  |  |  | 1 |
| STK19P | X |  |  |  |  | 1 |
| FKBPL |  | X |  |  |  | 1 |
| LINC00243 |  | X |  |  |  | 1 |
| HLA-DRB9 |  | X |  |  |  | 1 |
| SKIV2L |  | X |  |  |  | 1 |
| PBX2 |  | X |  |  |  | 1 |
| CLIC1 |  | X |  |  |  | 1 |
| IER3 |  | X |  |  |  | 1 |
| FLOT1 |  | X |  |  |  | 1 |
| LTB |  |  | X |  |  | 1 |
| PPP1R10 |  |  |  | X |  | 1 |
