# Supplementary material for: Genetic Association Reveals Protection against Recurrence of Clostridium difficile Infection with Bezlotoxumab Treatment
Source: mSphere. 2020 May 6;5(3):e00232-20. doi: 10.1128/mSphere.00232-20 (PMC7203456; doi:10.1128/mSphere.00232-20)
Supplement: TABLE S3 [file mSphere.00232-20-st003.docx]

| Gene | eQTL  (GTex V7)(3) | eQTL  (Chen 2016)(4) | eQTL  (Fairfax 2014)(5) | meth-QTL  (Chen 2016)(4) | pQTL  (Sun 2018)(6) |
| --- | --- | --- | --- | --- | --- |
| *MICB* | ↓ Adipose (visceral omentum)  ↓ Skin (not sun exposed)  ↓ Skin (sun exposed)  ↓ Thyroid | ↓ Naive CD4+ T cells  ↑ CD14+ monocytes |  | ↑ CD16+ neutrophils | ↑ Plasma |
| *HCG27* | ↑ Esophagus mucosa | ↑ Naive CD4+ T cells  ↑ CD14+ monocytes  ↑ CD16+ neutrophils |  |  |  |
| *C4B* |  | ↓ Naive CD4+ T cells |  |  | ↑ Plasma |
