# Supplementary material for: Genetic Association Reveals Protection against Recurrence of Clostridium difficile Infection with Bezlotoxumab Treatment
Source: mSphere. 2020 May 6;5(3):e00232-20. doi: 10.1128/mSphere.00232-20 (PMC7203456; doi:10.1128/mSphere.00232-20)
Supplement: TABLE S4 [file mSphere.00232-20-st004.docx]

| Characteristic | SNP+ | SNP- | HLA+ | HLA- |
| --- | --- | --- | --- | --- |
| N | 407 | 591 | 199 | 784 |
| Age, mean (SD) | 61.4 (16.9) | 62.2 (17.8) | 62 (16.2) | 62 (17.8) |
| Age, median | 64 | 64 | 64 | 64 |
| Age, range | 19–99 | 18–97 | 18–95 | 18–99 |
| Sex, female | 242 (59.5) | 353 (59.7) | 125 (62.8) | 464 (59.2) |
| ≥65 years of age | 193 (47.4) | 294 (49.7) | 98 (49.2) | 384 (49.0) |
| ≥1 CDI episodes in past 6 months | 122 (30.0) | 162 (27.4) | 51 (25.6) | 229 (29.2) |
| ≥2 previous CDI episodes ever | 69 (17.0) | 100 (16.9) | 34 (17.1) | 130 (16.6) |
| Severe CDI (Zar score ≥2)^a^ | 46 (11.3) | 75 (12.7) | 24 (12.1) | 97 (12.4) |
| Immunocompromised | 61 (15.0) | 109 (18.4) | 22 (11.1) | 144 (18.4) |
| Charlson Comorbidity Index ≥3 | 135 (33.2) | 224 (37.9) | 65 (32.7) | 287 (36.6) |
| Albumin ≤2.5 g/dl | 35 (8.6) | 69 (11.7) | 16 (8.0) | 87 (11.1) |
| Ribotype 027, 078, or 244 | 46 (17.7) | 73 (19.8) | 25 (19.2) | 91 (18.5) |
| Antibiotic use during ADT | 110 (27.0) | 185 (31.3) | 52 (26.1) | 240 (30.6) |
| Antibiotic use after ADT | 101 (24.8) | 174 (29.4) | 57 (28.6) | 213 (27.2) |
| ≥1 risk factor for rCDI^b^ | 282 (69.3) | 421 (71.2) | 133 (66.8) | 561 (71.6) |
