# Supplementary material for: Genetic Association Reveals Protection against Recurrence of Clostridium difficile Infection with Bezlotoxumab Treatment
Source: mSphere. 2020 May 6;5(3):e00232-20. doi: 10.1128/mSphere.00232-20 (PMC7203456; doi:10.1128/mSphere.00232-20)
Supplement: TABLE S5 [file mSphere.00232-20-st005.docx]

|  | Total population | | | | |  | Genetically defined Caucasian population | | | | |
| --- | --- | --- | --- | --- | --- | --- | --- | --- | --- | --- | --- |
| Genotype 🡪  *[population rel. freq.]* | CC  *[.59]* | TC  *[.35]* | TT  *[.06]* | TC or TT *[.41]* | Overall |  | CC  *[.57]* | TC  *[.37]* | TT  *[.07]* | TC or TT  *[.43]* | Overall |
| BEZ and BEZ+ACT | 31.8%  (87/274) | 12.7%  (20/157) | 3.3%  (1/30) | 11.2%  (21/187) | 23.4%  (108/461) |  | 32.1%  (68/212) | 13.3%  (16/120) | 2.9%  (1/34) | 11.0%  (17/154) | 23.2%  (85/366) |
| PBO | 35.3%  (48/136) | 32.2%  (29/90) | 35.7%  (5/14) | 32.7%  (34/104) | 34.2%  (82/240) |  | 37.2%  (42/113) | 32.5%  (27/83) | 36.4%  (4/11) | 33.0%  (31/94) | 35.3%  (73/207) |
| Risk difference | -3.5% | -19.5% | -32.4% | -21.5% | -10.7% |  | -5.1% | -19.2% | -33.4% | -21.9% | -12.0% |
| Relative risk | 0.90 | 0.40 | 0.09 | 0.34 | 0.69 |  | 0.86 | 0.41 | 0.08 | 0.33 | 0.66 |
