# Supplementary material for: Genetic Association Reveals Protection against Recurrence of Clostridium difficile Infection with Bezlotoxumab Treatment
Source: mSphere. 2020 May 6;5(3):e00232-20. doi: 10.1128/mSphere.00232-20 (PMC7203456; doi:10.1128/mSphere.00232-20)
Supplement: TABLE S6 [file mSphere.00232-20-st006.docx]

|  | Total population | | | | |  | Genetically defined Caucasian population | | | | |
| --- | --- | --- | --- | --- | --- | --- | --- | --- | --- | --- | --- |
| Genotype 🡪  *[population rel. freq.]* | X:X  *[.81]* | 0701:X  *[.18]* | 0701:0701  *[.01]* | 0701:X or 0701:0701  *[.19]* | Overall |  | X:X  *[.79]* | 0701:X  *[.20]* | 0701:0701  *[.01]* | 0701:X or 0701:0701  *[.21]* | Overall |
| BEZ and BEZ+ACT | 27.3%  (101/370) | 6.4%  (5/78) | 0.0%  (0/5) | 6.0%  (5/83) | 23.4%  (106/453) |  | 26.0%  (79/304) | 6.7%  (5/75) | 0%  (0/4) | 6.3%  (5/79) | 21.9%  (84/383) |
| PBO | 32.8%  (62/189) | 38.6%  (17/44) | 33.3%  (1/3) | 38.3%  (18/47) | 33.9%  (80/236) |  | 34.8%  (56/161) | 38.1%  (16/42) | 50.0%  (1/2) | 38.6%  (17/44) | 39.0%  (80/205) |
| Risk difference | -5.5% | -32.2% | -33.3% | -32.3% | -10.5% |  | -8.8% | -31.4% | -50% | -32.3% | -17.1% |
| Relative risk | 0.83 | 0.27 | 0.0 | 0.16 | 0.69 |  | 0.75 | 0.18 | 0 | 0.16 | 0.56 |
