# Supplementary material for: Genetic Association Reveals Protection against Recurrence of Clostridium difficile Infection with Bezlotoxumab Treatment
Source: mSphere. 2020 May 6;5(3):e00232-20. doi: 10.1128/mSphere.00232-20 (PMC7203456; doi:10.1128/mSphere.00232-20)
Supplement: TABLE S7 [file mSphere.00232-20-st007.docx]

| Genotype 🡪  *[population rel. freq.]* | CC or X:X  *[.55]* | TC or TT or 0701:X or 0701:0701  *[.45]* | Overall | (TC or TT) & (0701:X or 0701:0701):  N = 102 |
| --- | --- | --- | --- | --- |
| BEZ and BEZ+ACT | 33.1%  (86/260) | 11.3%  (23/204) | 23.5%  (109/464) | 4.5%  (3/66) |
| PBO | 32.0%  (40/125) | 36.5%  (42/115) | 34.2%  (82/240) | 27.8%  (10/36) |
| Risk difference | 1.1% | -25.2% | -10.7% | -23.2% |
| Relative risk | 1.03 | 0.31 | 0.69 | 0.16 |
