# Supplementary material for: Genetic Association Reveals Protection against Recurrence of Clostridium difficile Infection with Bezlotoxumab Treatment
Source: mSphere. 2020 May 6;5(3):e00232-20. doi: 10.1128/mSphere.00232-20 (PMC7203456; doi:10.1128/mSphere.00232-20)
Supplement: TEXT S1 [file mSphere.00232-20-s0001.docx]

# Supplemental Methods

## GWAS quality control

The MODIFY I and MODIFY II genotype data were genotyped using the Merck custom (Affymetrix) Axiom chip with BSA as the genotyping vendor. We followed the best practices guidelines provided by Affymetrix. All genotype data were presented as the forward (+) strand. The GWAS variant-level and subject-level QC workflows are summarized in Fig. S1. QC steps were done separately for MODIFY I and MODIFY II studies before SNP imputation. The following criteria were used for the main variant-level and subject-level QC steps.

- Exclude SNPs with HWE *P*-value < 1e-06.
  - Calculate HWE *P*-value using “—hardy” command in PLINK.
- Exclude variants with call rate < 97%.
  - Calculate missingness rates using “—missing” command in PLINK.

The following criteria were used for the main subject-level QC steps.

- Exclude individuals with dish QC < 0.82.
  - This is a standard Affymetrix experiment-related QC procedure recommended by the manufacturer.
- Exclude individuals with < 97% call rate.
  - Use “–missing” command in PLINK to recalculate missingness rates after variant filtering.
- Exclude subjects with mismatched genders.
  - Check concordance between reported gender in clinical database and gender derived from genetic data.
  - Use “–check-sex” command in PLINK to infer gender from genetic data.
- Exclude subjects with excessive heterozygosity rate.
  - Obtain the observed and expected homozygous calls using the “—het” command in PLINK. Using the resulting *.het file, calculate per sample heterozygosity (=(N_nonmissingGeno – N_homozygous)/N_nonmissingGeno), stratified by self-reported ethnicity. Individuals with +/-3*SD (standard deviation) from the mean in each stratum should be excluded.
- Exclude related individuals.
  - Use “–genome” command in PLINK to estimate pairwise identity by descent (IBD) between the subjects.
  - Identify pairs with PI_HAT > 0.1875. Generate a minimal list of individuals to remove with leave-one-out strategy.

## SNP imputation

The details of the SNP imputation and the HLA imputation are as follows.

- Imputation was performed separately in each ethnic group using 1000G Phase 1 reference panel using IMPUTE2.
  - The SNPs with low imputation quality (*r*^2^ < 0.3) were removed after imputation.
- HLA imputation was performed using HLA Genotype Imputation with Attribute Bagging (HIBAG).
  - The best-guess imputed HLA types were used while setting the call rate threshold to 0.5, meaning that the imputed genotypes were set as missing if their imputation posterior probability was less than 0.5 for three class I genes (*HLA-A*, *HLA-B*, *HLA-C*) and four class II genes (*HLA-DRB1*, *HLA-DQB1*, *HLA-DQA1*, *HLA-DPB1*).

## Principal Component Analysis (PCA)

The following steps were used to run the PCA analysis.

- Pool the data with 1000G Phase 3 genotypes.
- Perform PCA using EIGENSTRAT. Use option "-w poplist" to compute eigenvectors using only the 1000G super-populations (and study samples are projected onto the eigenvectors inferred from the 1000G populations).
- Plot PCs 1–5 one versus each other, highlighting the 1000G super populations (European, African, East Asian, South Asian, American) and the study population.
- Identify the clusters of study samples that overlap with the 1000G populations of interest and exclude the outliers and/or individuals whose self-reported ethnicity do not match with genetically identified ethnicity.
- Run a second PCA separately in each ethnic group of the study samples (without 1000G samples) to calculate PCs that will be used as covariates in the genetic studies.

The PCA analysis results are summarized in Fig. S2, which includes the scree plot and the pairwise scatter plots of the top four principal components (PCs).

# Replication of *rs4073* (Chr4:74606024)’s association with rCDI from Garey *et al*., 2010

Garey *et al,* (1) reported in their prospective cohort study that a common polymorphism *rs4073* (Chr4:74606024) in the interleukin-8 promoter region was associated with an increased risk for rCDI, with participants carrying the AA allele having an approximate two-fold greater risk of rCDI than participants with AT or TT genotypes.

Data from our PGx study show that, in the Caucasian population, SNP rs4073 (4:74606024, MAF 0.48) was not associated with a reduction in rCDI in bezlotoxumab-treated participants (p = 0.36) or in placebo-treated participants (p = 0.29). The bezlotoxumab P-value was estimated from the 2-df likelihood ratio test in a logistic regression model in both the treatment and placebo arms. The full statistical model and the comparative statistical model are the same as those in the Methods section in the main manuscript, except that the treatment term and the genotype by treatment interaction term should be dropped in the full statistical model and the treatment term should be dropped in the comparative statistical model. The placebo P-value was estimated from the 1-df likelihood ratio test in a logistic regression model in the placebo arm only.

Our results demonstrated that *rs4073* did not show an association with the rCDI in either baseline (or placebo) arm or with treatment arms, which is consistent with the conclusions from Miyajima *et al*., 2014 (2). In summary, our PGx data fails to replicate Garey *et al*., 2010’s association results in the interleukin-8 promoter region.

**References**

1. Garey KW, Jiang ZD, Ghantoji S, Tam VH, Arora V, Dupont HL. 2010. A common polymorphism in the interleukin-8 gene promoter is associated with an increased risk for recurrent Clostridium difficile infection. Clin Infect Dis 51:1406-1410.

2. Miyajima F, Swale A, Zhang JE, Alfirevic A, Little M, Beeching NJ, Smith G, Kolamunnage-Dona R, Pirmohamed M. 2014. Is the interleukin 8 promoter polymorphism rs4073/-251T >A associated with Clostridium difficile infection? Clinical infectious diseases : an official publication of the Infectious Diseases Society of America 58:e148-e151.

3. Consortium GT, Laboratory DA, Coordinating Center —Analysis Working G, Statistical Methods groups—Analysis Working G, Enhancing Gg, Fund NIHC, Nih/Nci, Nih/Nhgri, Nih/Nimh, Nih/Nida, Biospecimen Collection Source Site— N, Biospecimen Collection Source Site— R, Biospecimen Core Resource— V, Brain Bank Repository—University of Miami Brain Endowment B, Leidos Biomedical—Project M, Study E, Genome Browser Data I, Visualization—Ebi, Genome Browser Data I, Visualization—Ucsc Genomics Institute UoCSC, Lead a, Laboratory DA, Coordinating C, management NIHp, Biospecimen c, Pathology, e QTLmwg, Battle A, Brown CD, Engelhardt BE, Montgomery SB. 2017. Genetic effects on gene expression across human tissues. Nature 550:204-213.

4. Chen L, Ge B, Casale FP, Vasquez L, Kwan T, Garrido-Martín D, Watt S, Yan Y, Kundu K, Ecker S, Datta A, Richardson D, Burden F, Mead D, Mann AL, Fernandez JM, Rowlston S, Wilder SP, Farrow S, Shao X, Lambourne JJ, Redensek A, Albers CA, Amstislavskiy V, Ashford S, Berentsen K, Bomba L, Bourque G, Bujold D, Busche S, Caron M, Chen S-H, Cheung W, Delaneau O, Dermitzakis ET, Elding H, Colgiu I, Bagger FO, Flicek P, Habibi E, Iotchkova V, Janssen-Megens E, Kim B, Lehrach H, Lowy E, Mandoli A, Matarese F, Maurano MT, Morris JA, Pancaldi V, et al. 2016. Genetic Drivers of Epigenetic and Transcriptional Variation in Human Immune Cells. Cell 167:1398-1414.e24.

5. Fairfax BP, Humburg P, Makino S, Naranbhai V, Wong D, Lau E, Jostins L, Plant K, Andrews R, McGee C, Knight JC. 2014. Innate immune activity conditions the effect of regulatory variants upon monocyte gene expression. Science (New York, NY) 343:1246949-1246949.

6. Sun BB, Maranville JC, Peters JE, Stacey D, Staley JR, Blackshaw J, Burgess S, Jiang T, Paige E, Surendran P, Oliver-Williams C, Kamat MA, Prins BP, Wilcox SK, Zimmerman ES, Chi A, Bansal N, Spain SL, Wood AM, Morrell NW, Bradley JR, Janjic N, Roberts DJ, Ouwehand WH, Todd JA, Soranzo N, Suhre K, Paul DS, Fox CS, Plenge RM, Danesh J, Runz H, Butterworth AS. 2018. Genomic atlas of the human plasma proteome. Nature 558:73-79.
