# Supplementary figures and images for: Genetic Association Reveals Protection against Recurrence of Clostridium difficile Infection with Bezlotoxumab Treatment
Source: mSphere. 2020 May 6;5(3):e00232-20. doi: 10.1128/mSphere.00232-20 (PMC7203456; doi:10.1128/mSphere.00232-20)

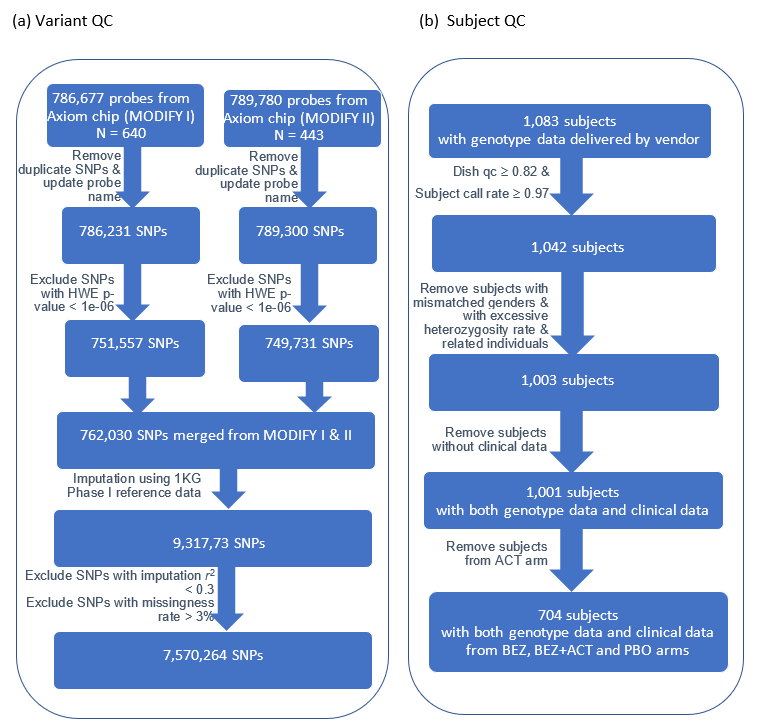

Supplement: FIG S1 [file mSphere.00232-20-sf001.docx]

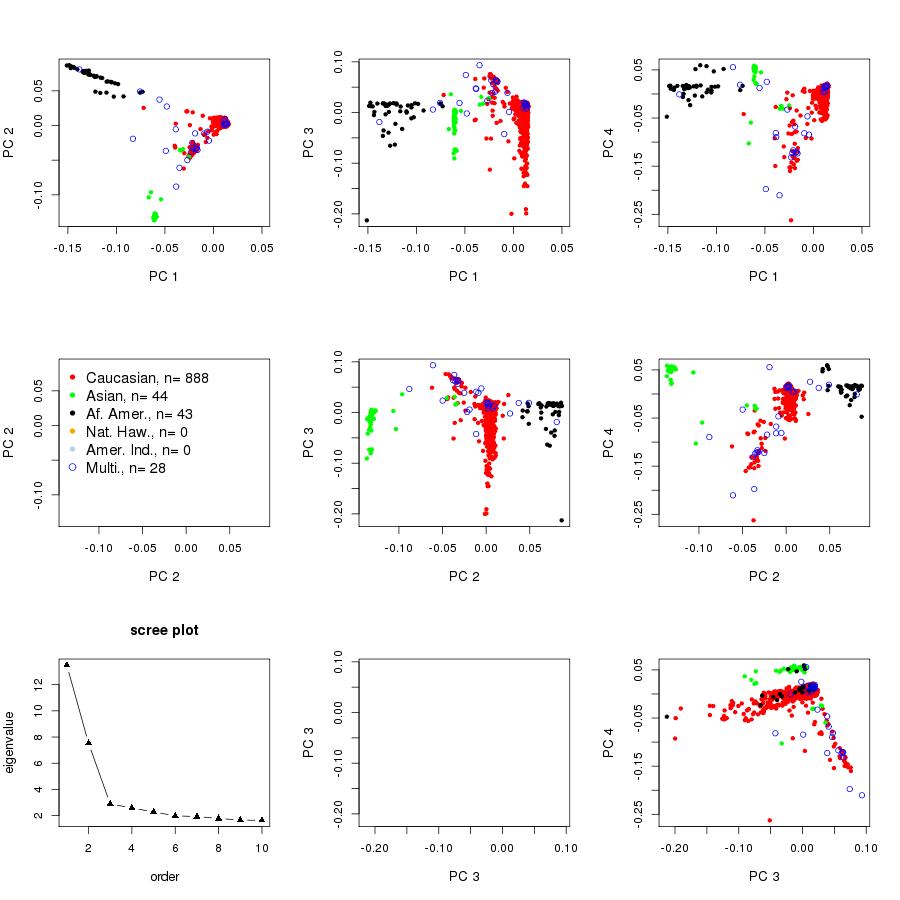

Supplement: FIG S2 [file mSphere.00232-20-sf002.docx]
